# Predictors of impaired SARS-CoV-2 immunity in healthcare workers after vaccination with BNT162b2

**DOI:** 10.1038/s41598-022-10307-8

**Published:** 2022-04-14

**Authors:** Sebastian Bertram, Arturo Blazquez-Navarro, Maximilian Seidel, Bodo Hölzer, Felix S. Seibert, Adrian Doevelaar, Benjamin Rohn, Panagiota Zgoura, Alexandra Witte-Lack, Sarah Skrzypczyk, David Scholten, Klaus Kisters, Nina Babel, Timm H. Westhoff

**Affiliations:** 1 Medical Department 1, University Hospital Marien Hospital Herne, Ruhr-University Bochum, Hölkeskampring 40, 44625 Herne, Germany; 2 Center of Translational Medicine, University Hospital Marien Hospital Herne, Ruhr-University, Bochum, Germany; 3 Medical Department, Marien Hospital, Witten, Germany; 4 Medical Department, St. Anna Hospital, Herne, Germany

**Keywords:** Preventive medicine, Occupational health, Risk factors, Viral infection, Epidemiology

## Abstract

Healthcare workers are at substantially increased risk for infection with SARS-CoV-2. Successful vaccination constitutes a crucial prerequisite to protect this group during the pandemic. Since post vaccination antibody monitoring is not standard of care in all healthcare institutions, data on risk factors of impaired vaccine induced immune response are urgently required. Moreover, there are no data on cellular immune responses in humoral low responders so far. Anti-SARS-CoV-2 spike IgG was assessed after vaccination with BNT162b2 in 1386 employees of three hospitals of a German healthcare provider. Concentrations were compared to those of 45 convalescent employees. Vaccine-induced cellular immunity was measured in employees with reduced humoral response by assessment of frequencies of SARS-CoV-2-reactive CD4^+^ and CD8^+^ T cell. Anti-SARS-CoV-2 spike IgG were detected in 99.9% of 1386 healthcare workers after completed vaccination. The median antibody concentration was significantly higher after vaccination than after infection with SARS-CoV-2 (p = 0.0001). 10 subjects (0.7%) generated an IgG concentration < 100 IU/ml, and only two persons (0.1%, solid organ recipients) did not produce detectable antibodies at all. T cell responses of those subjects with submaximal or lacking humoral response were comparable to employees with maximal antibody titers. 50% of those individuals with impaired or lacking humoral immune response were on immunosuppression. Vaccination to SARS-CoV-2 with BNT162b2 is very effective in healthcare workers yielding a seroconversion rate of 99.9%. Immunosuppression is the most important risk factor of an impaired immune response. There was no case of vaccination failure without immunosuppression. Thus, post vaccination antibody monitoring is highly recommendable in those employees with immunosuppression.

## Introduction

Healthcare workers are at increased risk of infection with SARS-CoV-2 due to their high exposure to infected patients. Successful vaccination constitutes a crucial prerequisite to protect this group during the pandemic. BNT162b2 (Biontech, Pfizer), mRNA-1273 (Moderna) and ChAdOx1 (AstraZeneca) vaccines proved high efficacy and safety in approval studies^1–3^. BNT162b2 revealed an efficacy of 95%, mRNA-1273 of 94.1%, and ChAdOx1 of 70.4% in preventing Covid-19^1–3^. The epidemiology of healthcare workers is somewhat different, however, from the phase 3 study populations: whereas approval studies aim to cover the overall adult population including elderly subjects, healthcare workers are usually < 65 years and therefore have a lower level of comorbidities. Since post vaccination antibody monitoring is not standard of care in all healthcare institutions, data on risk factors of impaired vaccine induced immune response are urgently required. Identification of these risk factors would allow an individualized immune-monitoring concept focusing on healthcare workers with a high risk of low humoral response.

Whereas antibody testing for SARS-CoV-2 IgG has become broadly available, monitoring of SARS-CoV-specific cellular immunity is limited to specialized laboratories. It would be of high interest, whether healthcare workers with low vaccine-induced humoral immunity might evolve e robust SARS-CoV-2 specific T-cell immunity nevertheless. The possibility of generating a sufficient vaccine-induced T-cell response despite lacking B-cell reactivity has recently been described in patients with rituximab^4^. To date, there are not data on T-cell immunity in healthcare staff with low humoral immune response.

## Methods

### Population

We investigated antibody generation to SARS-CoV-2 wild type spike protein after vaccination with two doses of BNT162b2 in 1386 employees of three hospitals of a German healthcare provider and compared them to antibody concentrations of employees, who had previously been infected by SARS-CoV-2 (n = 45). Time between second vaccination or diagnosis of COVID-19 was > 2 weeks in all participants. Clinical data and data on vaccine-induced SARS-CoV-2 specific T-cell immunity were obtained in those healthcare workers with low humoral immune response as described below.

### Measurement of SARS-CoV-2 anti-spike IgG

The Elecsys® Anti-SARS-CoV-2 S, (Roche Diagnostics International Ltd, Rotkreuz, Switzerland) immunoassay was used for measurement of IgG to SARS-CoV-2 spike protein having a linear detection range up to 250 IU/ml. In accordance with previous large studies, we defined an arbitrary antibody concentrations < 100 IU/ml as “low humoral vaccine-induced immune response”^5^.

### Assessment of vaccine-induced SARS-CoV-2 specific T-cell immunity

SARS-CoV-2 specific T-cell immunity was assessed in 9 subjects with antibody concentrations < 100 IU/ml after vaccination and a in comparison group with healthcare workers with a maximal humoral response. There was no further selection in the comparison group. The number of analyses in maximal humoral (n = 12) response were comparable to those with limited humoral response (n = 9). Cellular immunity was monitored by assessment of frequencies of SARS-CoV-2–reactive CD4^+^ and CD8^+^ T cells as previously described^6^. Briefly, peripheral blood mononuclear cells (PBMC) were isolated from EDTA collection tubes (Sarstedt, Germany) and afterwards stimulated with SARS-CoV-2-PepTivator peptide-pools solved in water (Miltenyi Biotec, Germany). Untreated PBMC were used as negative control to assess unspecific background activation. After 2 h of stimulation, Brefeldin-A (Sigma-Aldrich, Germany) was added and the stimulation stopped after 16 h. Surface- and intracellular-staining for flow cytometry was performed using fixation and permeabilization (ThermoFisher, Germany) and antibodies including anti CD3, abti-CD45, anti-CD3, anti-CD4, anti-CD8, anti-IL-2, anti-IFNg, anti-IL4, anti-TNFa, anti-GrzB, anti-CD45RA, anti-CCR7. Samples were measured on a CytoFlex flow cytometer (Beckman-Coulter).

Flow cytometry data were analyzed using FlowJo version 10.6.2 (BD Biosciences). Single stains and fluorescence-minus-one controls were used for gating. CD4^+^ T cells expressing CD154 and CD137 and CD8^+^ T cells expressing CD137 in combination with production of at least one of cytokines were defined as reactive T cells. Unspecific activation in unstimulated controls was subtracted from stimulated samples to account for SARS-CoV-2-specific activation in the presented frequencies.

### Statistics

Data are presented as median and interquartile range (IQR). Mann–Whitney test was used to compare age and S^−^ reactive antibody titers between vaccinated and convalescent healthcare workers. Mann–Whitney was used to compare the CD4^+^ and CD8^+^ response of individuals with impaired humoral response and maximal humoral response. Fisher’s exact test was applied to analyze the gender distribution of vaccinated and convalescent healthcare workers. Differences in assignment to one of the antibody concentration strata was analyzed by Chi-squared test. p < 0.05 were considered statistically significant. Statistical analysis was performed with GraphPad Prism (Version 9.12, GraphPad Software, La Jolla California USA).

### Ethics approval and consent to participate

This study was conducted in accordance with the declaration of Helsinki and was approved by the ethics committee of Ruhr-University Bochum (20-6886; 20-7126). Informed consent was obtained from all participants.

## Results

**Figure 1:**
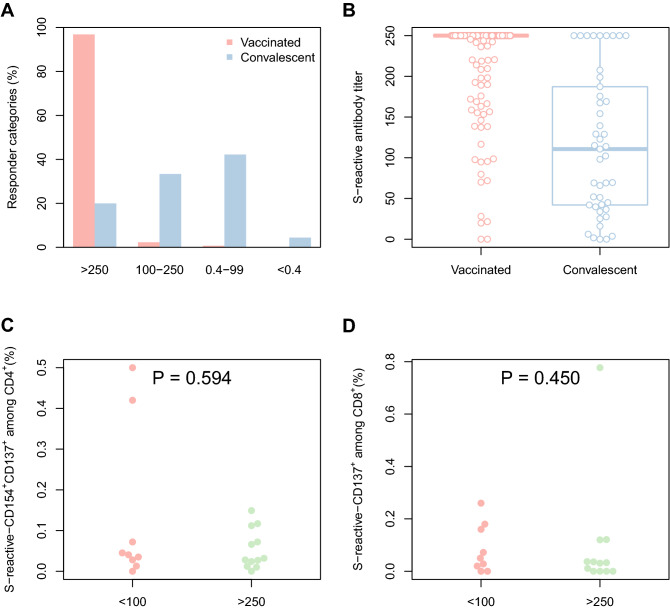
(**A**) Distribution of quantitative Anti-SARS-CoV-2 IgG response of vaccinated and convalescent healthcare workers. (**B**) S (spike protein) − reactive antibody titers of vaccinated (median 250, IQR 250–250) and convalescent (110.7, IQR 40.9–193.1, p = 0.001 Mann Whitney test) healthcare workers. (**C**) SARS-CoV-2 spike protein reactive CD4^+^ (p = 0.59) and (**D**) CD8^+^ (p = 0.45) T-cells in healthcare workers with anti-SARS-CoV-2 IgG < 100 U/ml vs. anti-SARS-CoV-2 IgG > 250 U/ml showing a comparable vaccine-induced T-cell response.

1386 healthcare workers agreed to undergo measurement of anti-SARS-CoV-2 spike protein after completed vaccination with BNT162b2 (assessment > 2 weeks after second dosage in all the employees). Table 1 summarizes epidemiology and humoral immunity in vaccinated and infected healthcare workers. As presented in Fig. 1, 1342 of 1386 employees (96.9%) developed the maximal detectable anti SARS-CoV-2 IgG concentration (> 250 IU/ml) after vaccination. A submaximal humoral immune response (SARS-CoV-2 IgG 100–250 IU/ml) was observed in n = 32 (2.3%). 10 subjects (0.7%) generated an IgG concentration < 100 IU/ml, and only two persons (0.1%) did not produce detectable antibodies at all. The median antibody concentration was significantly higher after vaccination (250 IQR 250–250) than after infection with SARS-CoV-2 (110.7 IQR 40.89–193.1, p = 0.0001).

**Table 1:** Epidemiology and SARS-CoV-2 reactive humoral immune response in healthcare workers after vaccination or prior COVID-19.

	Persons vaccinated by BNT162b2 (n = 1386)	Persons with prior SARS-CoV-2 infection, no vaccination (n = 45)	p
Median time after infection/vaccination (weeks)	8 (IQR 7–9)	20 (15–28)	< 0.0001
Median age (years)	45 (IQR 33–55)	33 (IQR 25–40.5)	0.0001
Female gender (n, %)	1025, 73.9%	27, 60%	0.04
Anti-SARS-CoV-2 IgG > 250 IU/ml (n, %)	1342, 96.9%	9, 20%	0.0001
Anti-SARS-CoV-2 IgG 100–250 IU/ml (n, %)	32, 2.3%	15, 33.3%	
Anti-SARS-CoV-2 IgG 0.4–99 IU/ml (n, %)	10, 0.7%	20, 44.5%	
Anti-SARS-CoV-2 negative (n, %)	2, 0.1%	1, 2.2%	

Data of vaccinated vs. convalescent healthcare workers were compared using Mann–Whitney test in case of continuous data, and by Fisher’s exact test in case of dichothomic data. Differences in assignment to one of the antibody concentration strata was analyzed by Chi-squared test.

T-cell immunity was investigated in those individuals with reduced humoral response (SARS-CoV-2 IgG < 100 IU/ml) and compared to 12 vaccinated subjects with maximal response (SARS-CoV-2 IgG > 250 U/ml). A SARS-CoV-2 reactive cellular immunity against the viral spike protein (S-reactive) was detected in nine of these healthcare workers. Thus, 88.9% and 77.8% of the probands exhibited a detectable CD4^+^ and CD8^+^ response, respectively. Among those with a detectable immune response, the frequency of activated cells was 0.04 [0.03–0.15]% among CD4^+^ and 0.07 [0.04–0.17]% among CD8^+^ T cells. Importantly, the magnitude of the observed T cell responses were comparable and revealed no significant difference to those subjects with maximal humoral immunity (Fig. 1C,D).

Infection with SARS-CoV-2 despite vaccination was documented in 3 persons > 2 weeks after the first vaccine dose and in 0 persons > 2 weeks after the second dose. All of these infections revealed an asymptomatic or mild course and were managed in an out-patient setting.

Subjects with low (< 100 IU/ml) or no generation of antibodies had a median age of 56 (IQR, 40.25–62.00) and a median body mass index of 24.34 kg/m^2^ (IQR, 21.63–27.75). Some of them suffered from at least one cardiovascular disease (21.4% hypertension, 8.3% diabetes, 8.3% coronary heart disease, 0% congestive heart failure). Six of these persons (50%) were on immunosuppressive medication (methotrexat, certolizumab, azathioprin, infliximab, leflunomid, tacrolimus, mycophenolate mofetile and prednisolone). Both healthcare workers without seroconversion were renal transplant recipients with triple immunosuppression (tacrolimus, mycophenolate mofetile and prednisolone). Thus, 50% of those individuals with impaired or lacking vaccination response were on immunosuppression.

## Discussion

BNT162b2 proved very high effectiveness in healthcare workers yielding a seroconversion rate of 99.8%. Interestingly, even in submaximal humoral SARS-CoV-2 reactive immune response, cellular immunity was robust. No severe infections occurred beyond 2 weeks after application of the second vaccine dose. Thus, vaccination to SARS-CoV-2 provides a high level of safety in this highly exposed population. Interestingly, antibody concentrations exceeded those after SARS-CoV-2 infection by far.

Immunosuppressive medication was identified as the main risk factor for impaired humoral immune response after vaccination in the healthcare population. Seroconversion failures occurred only in solid organ recipients. Regarding the humoral vaccination response > 99%, our findings are in line with a recently published report on BNT162b2 vaccination in health care workers^7^. In addition to this initial report, our study provides first data on vaccine-induced T-cell responses in this population and identifies immunosuppression as a risk factor for vaccination failure. Noteworthy, the vast majority of those individuals with limited humoral immune response had a T-cell response comparable to subjects with maximal antibody response.

This study has some implications for the vaccination management in healthcare staff: it appears highly recommendable to routinely assess SARS-CoV-2 antibodies in healthcare workers with immunosuppression. Compared to immunosuppression, cardiovascular diseases were of neglectable relevance for impaired immune response.

In our study, AK titers and T-cell response were determined after a short interval of infection or vaccination. This includes the limitation that a submaximal immune response can be assumed during this time. Further follow-up is necessary to assess the question of longer-term immune response. With regard to the high exposure to SARS-CoV-2, a booster vaccination by a third vaccine dose may be considered in case of documented vaccination failure in healthcare workers. There are first reports that demonstrate that a third vaccine dose yields seroconversion in solid organ transplant recipients with primary vaccination failure^8,9^. By means of this screening approach vaccination success is almost 100% in the highly exposed population of healthcare workers.
